# Assessment of solar radiation resource from the NASA-POWER reanalysis products for tropical climates in Ghana towards clean energy application

**DOI:** 10.1038/s41598-022-14126-9

**Published:** 2022-06-23

**Authors:** Alfred Dawson Quansah, Felicia Dogbey, Prince Junior Asilevi, Patrick Boakye, Lawrence Darkwah, Sampson Oduro-Kwarteng, Yen Adams Sokama-Neuyam, Patrick Mensah

**Affiliations:** 1 Research and Development Division, Ghana National Petroleum Corporation, Tema, Ghana; 2 Department of Mechanical Engineering, Kwame Nkrumah University of Science and Technology, Kumasi, Ghana; 3 Meteorology and Climate Science Unit, Department of Physics, Kwame Nkrumah University of Science and Technology, Kumasi, Ghana; 4 Department of Civil Engineering, Kwame Nkrumah University of Science and Technology, Kumasi, Ghana; 5 Department of Chemical Engineering, Kwame Nkrumah University of Science and Technology, Kumasi, Ghana; 6 Department of Petroleum Engineering, Kwame Nkrumah University of Science and Technology, Kumasi, Ghana; 7 Department of Mechanical Engineering, Southern University and A&M College, Baton Rouge, LA USA

**Keywords:** Ecology, Environmental sciences, Renewable energy

## Abstract

In order to expand the output of solar power systems for efficient integration into the national grid, solar energy resource assessment at site is required. A major impediment however, is the widespread scarcity of radiometric measurements, which can be augmented by satellite observation. This paper assessed the suitability of satellite-based solar radiation resource retrieved from the NASA-POWER archives at $0.5^\circ \times 0.5^\circ$ spatial resolution over Ghana–West Africa, to develop a long-term source reference. The assessment is based on the criteria of comparison with estimations from sunshine duration measurement for 22 synoptic stations. Overall, the satellite-based data compared well with ground-based estimations by r = 0.6–0.94 ± 0.1. Spatiotemporally, the agreement is strongest over the northern half Savannah-type climate during March–May, and weakest over the southern half Forest-type climate during June–August. The assessment provides empirical framework to support solar energy utilization in the sub-region.

## Introduction

Solar energy resource assessment is critical for accurate evaluation of the quantity of incoming solar radiation available to develop, install, and operationalize highly efficient solar power technologies^1,2^. The task primarily involves development of a comprehensive climatological solar radiation and related parameters database at short-time intervals, an in-depth grasp of the spatiotemporal distribution and correlations, and accurate forecasts to ascertain precisely the performance of solar power systems and the technical effect of solar radiation variability on national electric grids. However, due to sparse or non-existent solar radiation measurement stations in many parts of the world, the gap between installation and performance modeling widens^3–6^.

In the last recent decade, retrieving satellite products for solar energy resource assessment has become a state-of-the-art technique to bridge the gap of insufficient or non-existent solar radiation measurement stations^3,4,7^, especially as satellite products have the advantage of wide coverage^8–10^. Many studies have used satellite-based observations to predict solar radiation and develop estimation models especially in areas where insolation measurement stations are completely non-existent, towards efficient power generation^4,8,10–13^. However, despite the promising potentials of wide-coverage and quick-time earth observations associated with satellite-based datasets, a major application challenge immediately confronting the scientific community is resolution and local climate-specific representation viz. data accuracy. For example, Dubovik^14^ and Kim^15^ have clearly identified the limitation of high-orbit geostationary satellites in capturing surface phenomena at high resolution despite the advantage of wide spatial coverage over their Low-Earth Orbit satellite counterparts. Thus generally, wider spatial capture results in poor spatial resolution. Owing to this, reliance on satellite-based observations require synergistic support from ground data in the form of validation, merging, and reanalysis^14^. In this regard, Brinckmann^8^ reported a comprehensive assessment solar radiation products for Germany, generated from near-real-time satellite data retrieved from the Spinning Enhanced Visible and Infrared Imager (SEVIRI) onboard METEOSAT. The study involved comparison of the satellite-derived radiation data with measurements from 42 weather stations, and merging of the two datasets to develop a high spatial resolution hourly solar radiation repository for Germany. Yeom^10^ as well, assessed physical models based on imagery derived from the Communication, Ocean, and Meteorological Satellite (COMS), to estimate and develop solar energy maps for slops on the Korean peninsula, needed to demarcate potential sites for solar power plants. Other studies by Ghimire^4^ investigated the suitability of satellite-derived solar radiation data for the training of machine learning algorithms to predict solar resource for regional assessment.

Over the years, the National Aeronautics and Space Administration (NASA) has made significant efforts to improve satellite atmospheric observation data on a global scale, especially tailored to the needs of the renewable energy industry^16,17^. For example, development of the Surface Meteorological and Solar Energy (SSE) climatological resource database needed by the photovoltaic and renewable energy industries, was especially targeted at optimizing electric power from renewable energy systems, and ultimately towards the successful integration of the technology into conventional power generation systems^16,17^. Currently, under the NASA Science Mission Directorate Applied Science National Application program, the Prediction of Worldwide Energy Resource (POWER) project has been initiated to further improve the SSE database, augment with products retrieved from new satellites, and develop forecast models to serve specifically as decision support tools for the energy sector^18,19^. Due to the global coverage and accessibility, suitability assessment for regional application is needful. Notable effort include the report by Monteiro^20^ in the assessment of NASA-POWER solar radiation and weather related parameters for agricultural application in Brazil. The study found low root mean square error (RMSE) values for comparisons with station measurements. Marzouk^21^ also assessed NASA-POWER datasets to investigate climate change by global warming, compared with the output from of ground-based measurements. Looking forward, the NASA-POWER project is expected to expand with strong stakeholder involvement and industry support^16,20–22^.

With this background, the objective of this paper is to assess the suitability of the NASA-POWER Global solar radiation (GSR) products over Ghana in West Africa, which is expected to provide a comprehensive high density network GSR database for a solar resource assessment, as currently no regularized radiometric measurement stations are reported. Firstly, 35 years sunshine duration measurement datasets were used to estimate and develop a climatological monthly mean GSR dataset, in order to provide a statistical comparative reference for the NASA-POWER climatological GSR datasets. The study will serve a valuable tool to further develop forecasting models for performance enhancement of solar technologies.

## Methodology

### Geography and climatology of study area

**Figure 1:**
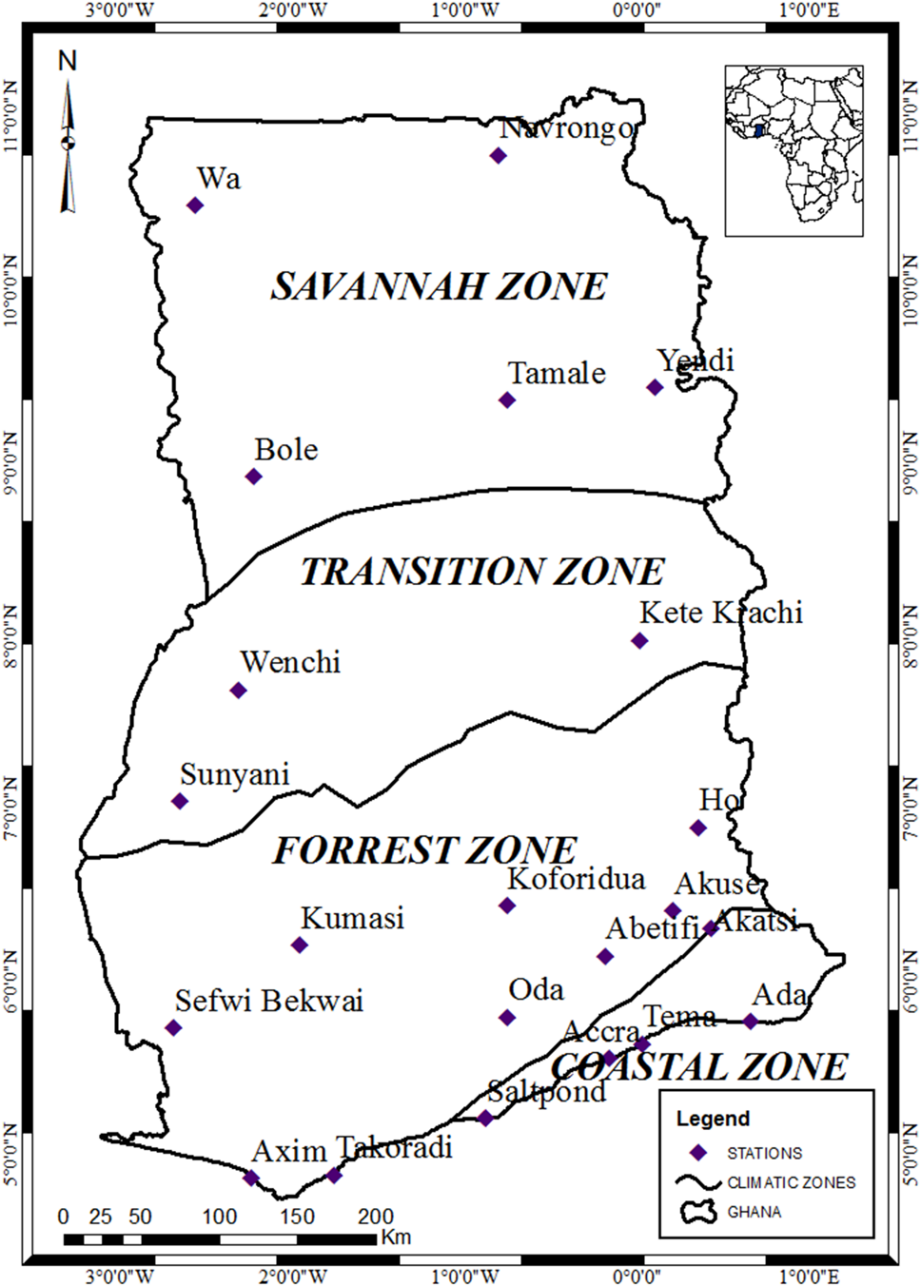
Map of the study area showing all twenty two (22) synoptic stations distributed in four main climatological zones countrywide. Adapted from Asilevi^27^.

The area of study, Ghana, is on the coastal edge of tropical West African, bounded in latitude 4.5° N and 11.5° N and longitude 3.5° W and 1.5° E, and characterized by a tropical monsoon climate system^23,24^. Figure 1 shows map of the study area indicating the selected twenty two (22) sunshine measurement stations distributed across the four main climatological zones and Table 1 summarizes the geographical positions of selected stations.

**Table 1:** Geographical position and elevation for study sites.

Station	Latitude (°)	Longitude (°)	Elevation (m)
Wa	10.05	− 2.50	305.00
Navrongo	10.90	− 1.10	197.00
Bole	9.33	− 2.48	246.94
Tamale	9.42	− 0.85	152.00
Yendi	9.45	− 0.17	157.00
Wenchi	7.75	− 2.10	299.00
Sunyani	7.33	− 2.33	305.00
Kete Krachi	7.82	− 0.33	92.00
Kumasi	6.72	− 1.60	256.00
Sefwi Bekwai	6.20	− 2.33	186.50
Oda	5.93	− 0.98	151.00
Abetifi	6.67	− 0.75	601.00
Koforidua	6.83	− 0.25	199.00
Ho	6.60	0.47	154.00
Akuse	6.10	0.12	107.63
Axim	4.90	− 2.25	71.00
Takoradi	4.88	− 1.77	74.00
Salt Pond	5.20	− 1.67	77.00
Accra	5.60	− 0.17	91.00
Tema	5.62	0.00	79.00
Ada	5.78	0.63	15.29
Akatsi	6.12	0.80	66.48

Atmospheric clarity over the area is closely connected to cloud amount distribution and rainfall activities, largely determined by the oscillatory migration of the Inter-Tropical Discontinuity (ITD), accounting for the West African Monsoon (WAM)^25,26^.

Owing to the highly variable spatiotemporal distribution of cloud amount vis-à-vis rainfall activities, resulting in contrasting climatic conditions in different parts of the region, the country is partitioned by the Ghana Meteorological Agency (GMet) into four main agro-ecological zones namely, the Savannah, Transition, Forest and Coastal zones as shown in Fig. 1^23^. As a result, the region experiences an estimated Global solar radiation (GSR) intensity peaks in April–May and then in October–November, with the highest monthly average of 22 MJm^−2^ day^−1^ over the savannah climatic zone and the lowest monthly average of 13 MJm^−2^ day^−1^ over the forest climatic zone^27^.

### Research datasets

#### Ground-based measurement data

Daily sunshine duration measurement datasets (n) spanning 1983–2018 where derived for estimating Global solar radiation (GSR). The measurements were taken by the Campbell-Stokes sunshine recorder, mounted at the 22 stations shown in Fig. 1, under unshaded conditions to ensure optimum sunlight exposure. The device concentrates sunlight onto a thin strip of sunshine card, which causes a burnt line representing the total period in hours during which sunshine intensity exceeds 120.0 Wm^−2^ according to World Meteorological Organization (WMO) recommendations^27^. The as-received daily records were quality control checked by ensuring 0 ≤ n ≤ N, where N is the astronomical day length representing the possible maximum duration of sunshine in hours determined by Eq. 1 from the latitude (ϕ) of the site of interest and the solar declination (δ) computed by Eq. 2^27^:

1 $$ {\text{N}} = \frac{2}{15}\cos^{ - 1} \left[ { - \tan \phi \tan {\updelta }} \right] $$

2 $$ {\updelta } = 23.45\sin \left[ {360^{{\text{o}}} \times \frac{{284 + {\text{J}}}}{365}} \right] $$

where J represents the number for the Julian day of the year (first January is 1 and second January is 2).

#### NASA-POWER Global solar radiation (GSR) reanalysis data

The satellite-based Global solar radiation (GSR) dataset for specific longitudes and latitudes of all 22 stations, assessed in the study, were retrieved from the National Aeronautics and Space Administration-Prediction of Worldwide Energy Resources (NASA-POWER) reanalysis repository based on the Modern Era Retrospective-Analysis for Research and Applications (MERRA-2) assimilation model products, developed from Surface Radiation Budget, and spanning equal study period (1983–2018). The datasets are accessible on a daily and monthly temporal resolution scales at 0.5° × 0.5° spatial coverage via a user friendly web-based mapping portal: https://power.larc.nasa.gov/data-access-viewer/^17^. The advantage of the NASA-POWER reanalysis GSR, is the wide spatial coverage, and thus can be used to develop a high spatial resolution of solar radiation across the study area.

The POWER Project analyzes, synthesizes and makes available surface radiation related parameters on a global scale, primarily from the World Climate Research Programme (WCRP), Global Energy and Water cycle Experiment (GEWEX), Surface Radiation Budget (SRB) project (Version 2.9), the Clouds and the Earth’s Radiant Energy System (CERES), FLASHFlux (Fast Longwave and Shortwave Radiative Fluxes from CERES and MODIS), and the Global Modeling and Assimilation Office (GMAO)^17^. Table 2 shows the source satellites and the corresponding temporal coverage used in the development of NASA-POWER GSR products.

**Table 2:** Satellites providing the NASA-POWER GSR datasets^20^.

Satellite	Time coverage
GEWEX SRB R4-IP	January 1, 1983 to December 31, 2000
CERES SYN1deg	January 1, 2001 to few months within
FLASHFlux 4	January 1, 2008 to near-real time

The monthly average NASA-POWER all-sky shortwave surface radiation reanalysis products are statistically validated, showing reasonable biases of − 6.6–13%, against a global network of surface radiation measurement metadata in an integrated database from the Baseline Surface Radiation Network (BSRN) of the World Radiation Monitoring Center (WRMC)^20,22^. The datasets are widely used in renewable energy application^16,22^, agricultural modelling of crop yields^28^, crop simulation exercises^29^, and plant disease modelling^30^.

Furthermore, in order to assess the suitability of the NASA-POWER surface solar radiation products for the study area, a synthetic sunshine duration based Global solar radiation (GSR) is developed from the Angstrom-Prescott sunshine duration model by Eq. 3 for comparisons^27^.

3 $$ {\text{GSR}} = \left[ {{\text{a}} + {\text{b}}\frac{{\text{n}}}{{\text{N}}}} \right]{\text{H}}_{{\text{o}}} $$

were H_o_ (kWhm^−2^ day^−1^) is the daily extraterrestrial solar radiation on an horizontal surface, n is the daily sunshine duration measurements obtained from the Ghana Meteorological Agency (GMet), and N is the maximum possible daily sunshine duration or the day length in hours determined by Eq. 1. Generalized regression constants a = 0.25 and b = 0.5 for the study area were determined by Asilevi^27^ from experimental radiometric data based on correlation regression analysis between atmospheric clarity index (GSR/H_o_) and atmospheric cloudlessness index (n/N), for estimating solar radiation over the study area, and compared with other satellite data retrieved from the National Renewable Energy Laboratory (NREL) and the German Aerospace Centre (DLR)^27^. H_o_ was calculated from astronomical parameters by Eq. 4:

4 $$ {\text{H}}_{0} = \frac{{24{ } \cdot { }60}}{\pi } \cdot {\text{G}}_{{{\text{sc}}}} \cdot {\text{d}}_{{\text{r}}} \left[ {\omega_{{\text{s}}} \sin \varphi \sin \delta + \cos \varphi \cos \delta \sin \omega_{{\text{s}}} } \right] $$

where G_sc_ is the Solar constant in MJm^−2^ min^−1^, d_r_ is the relative Earth–Sun distance in meters (m), $\omega_{s}$ is the sunset hour angle (angular distance between the meridian of the observer and the meridian whose plane contains the sun), $\delta$ is the angle of declination in degrees (°) and $\varphi$ is the local latitude. A detailed presentation of the calculation was published in a previous work^27^.

### Statistical assessment analysis

For the purpose of assessing the NASA-POWER derived monthly mean GSR (GSR_n_) datasets in comparison with the estimated Global Solar Radiation (GSR_e_) datasets used in this paper, the following deviation and correlation methods in Eqs. 5–11, each showing a complimentary result were used: Standard deviation (${\upsigma }$), residual error (RE), Root mean square error (RMSE), Mean bias error (MBE), Mean percentage error (MPE), Pearson’s correlation coefficient (r), and Willmott index of agreement (d) for n observations^31–35^. GSR_e_, GSR_n,_ and RE represent the estimated GSR, NASA-POWER GSR, and the residual error between GSR_e_ and GSR_n_ respectively. A positive RE indicates that sunshine-based estimated GSR is larger than the NASA-POWER reanalysis dataset, while a negative RE indicates that sunshine-based estimated GSR is smaller than the NASA-POWER reanalysis dataset. The arithmetic mean of any dataset is µ.

The standard deviation (${\upsigma }$) was used to check the upper and lower limits of distribution around the mean deviations between GSR_e_ and GSR_n_ in order to ascertain violations between both datasets^33^. The RMSE is a standard statistical metric to quantify error margins in meteorology and climate research studies, and by definition is always positive, representing zero in the ideal case, plus a smaller value signifying a good marginal deviation^31^. The MBE is a good indicator for under-or overestimation in observations, with MBE values closest to zero being desirable. The MPE further indicates the percentage deviation between the GSR_e_ and GSR_n_ individual datasets^35^.

5 $$ {\upsigma } = \sqrt {\frac{1}{{{\text{n}} - 1}}\mathop \sum \limits_{{{\text{i}} = 1}}^{{\text{n}}} \left( {{\text{GSR}} - {\upmu }} \right)^{2} } $$

6 $$ {\text{RE}} = {\text{GSR}}_{{\text{e}}} - {\text{GSR}}_{{\text{n}}} $$

7 $$ {\text{RMSE}} = \sqrt {\frac{1}{{\text{n}}}\mathop \sum \limits_{{{\text{i}} = 1}}^{{\text{n}}} \left( {{\text{RE}}} \right)^{2} } $$

8 $$ {\text{MBE}} = \frac{1}{{\text{n}}}\mathop \sum \limits_{{{\text{i}} = 1}}^{{\text{n}}} \left( {{\text{RE}}} \right) $$

9 $$ {\text{MPE}} = \frac{1}{{\text{n}}}\mathop \sum \limits_{{{\text{i}} = 1}}^{{\text{n}}} \left( {\frac{{{\text{RE}}}}{{{\text{GSR}}_{{\text{e}}} }} \times 100{\text{\% }}} \right) $$

10 $$ {\text{r}} = \frac{{\mathop \sum \nolimits_{{{\text{i}} = 1}}^{{\text{n}}} \left( {{\text{GSR}}_{{\text{e}}} - {\upsigma }_{{\text{e}}} } \right)\left( {{\text{GSR}}_{{\text{n}}} - {\upsigma }_{{\text{n}}} } \right)}}{{\left( {{\text{n}} - 1} \right){\upsigma }_{{\text{e}}} {\upsigma }_{{\text{n}}} }} $$

11 $$ {\text{d}} = 1 - \left[ {\frac{{\mathop \sum \nolimits_{{{\text{i}} = 1}}^{{\text{n}}} \left( {{\text{GSR}}_{{\text{e}}} - {\text{GSR}}_{{\text{n}}} } \right)^{2} }}{{\mathop \sum \nolimits_{{{\text{i}} = 1}}^{{\text{n}}} \left( {\left| {{\text{GSR}}_{{\text{e}}} - {\text{GSR}}_{{{\text{nave}}}} \left| + \right|{\text{GSR}}_{{\text{n}}} - {\text{GSR}}_{{{\text{nave}}}} } \right|} \right)^{2} }}} \right] $$

Further, as with other statistical studies in meteorology^36^, the Pearson’s correlation coefficient (r) was used to quantify the strength of correlation between GSR_e_ and GSR_n_. Finally, the Willmott index of agreement (d) commonly used in meteorological literature computed from Eq. 7 is used to assess the degree of GSR_e_/GSR_n_ agreement^34^.

## Results and discussions

### Comparison of estimated GSR and NASA-POWER satellite-based GSR

**Figure 2:**
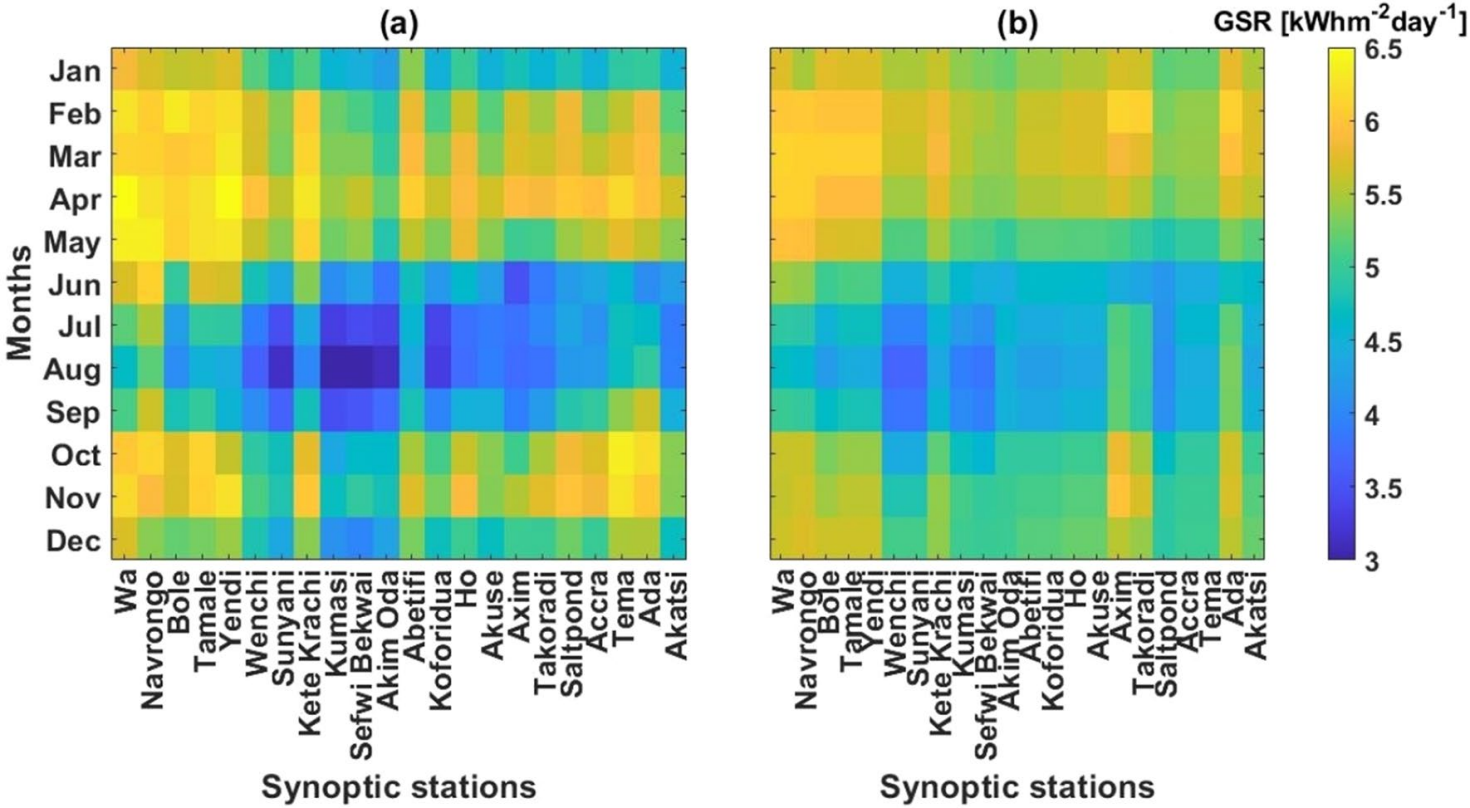
Space and time distribution of (**a**) estimated monthly mean Global Solar Radiation (GSR_e_) and (**b**) NASA-POWER derived monthly mean GSR (GSR_n_).

This section compared the time and space variations of climatological estimated monthly mean Global Solar Radiation (GSR_e_) and the NASA-POWER Global Solar Radiation (GSR_n_) datasets across the study area, in order to ascertain similarities. From Fig. 2, it is seen that both GSR_e_ (Fig. 2a) and GSR_n_ (Fig. 2b) show similarity is spatiotemporal distribution countrywide. That is peak insolation between February and May (GSR_e_ = 5.5–5.7 kWhm^−2^ day^−1^; GSR_n_ = 5.3–5.7 kWhm^−2^ day^−1^) with the highest over the Savannah climatic zone, and lowest insolation during the June–September season (GSR_e_ = 4.4–4.9 kWhm^−2^ day^−1^; GSR_n_ = 4.4–4.7 kWhm^−2^ day^−1^), the Forest climatic zone receiving the least in both datasets. Therefore even with slight variations, both datasets show significantly very good spatiotemporal arrangements countrywide. However on a daily scale comparison, relatively higher variations may be expected largely due to the complexities in atmospheric dynamics which have not been well parameterized in the observation satellite sensor^14,15^.

**Figure 3:**
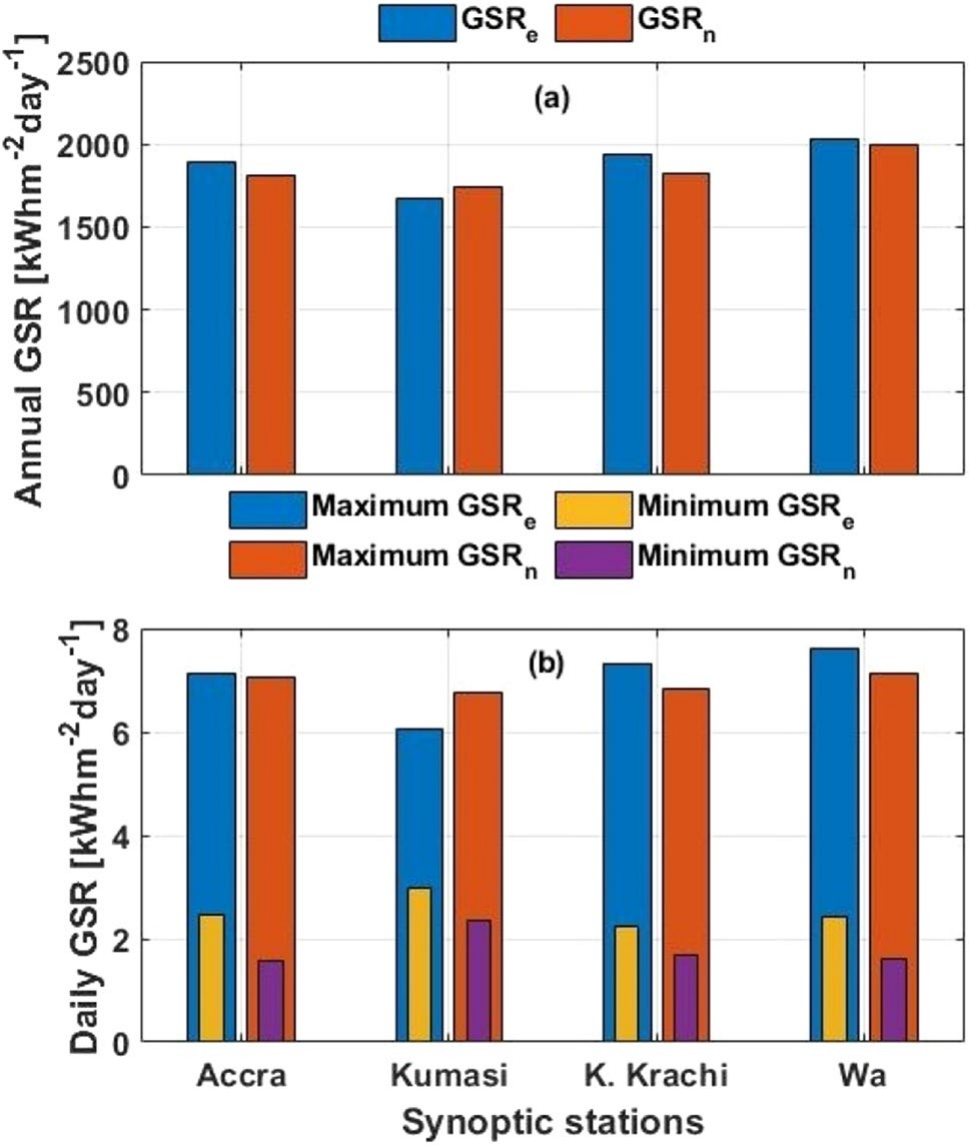
Comparing estimated and NASA-POWER GSR in 2018 on (**a**) annual total and (**b**) daily scales at four stations (Accra, Kumasi, Kete Krachi, and Wa) representing the Coastal, Forest, Transition, and Savannah climatic zones respectively.

In a further comparison, Fig. 3 shows the total annual GSR and daily maximum-minimum GSR for both datasets in 2018 at four (4) synoptic stations (Accra, Kumasi, Kete Krachi, and Wa) representative of the Coastal, Forest, Transition, and Savannah climatic zones respectively. As seen in Fig. 3a, the variation range in annual GSR for both datasets for all four stations is 33.1–109 kWhm^−2^ day^−1^, with only the Forest climatic zone showing slight over-estimation in the satellite data.

Again with Fig. 3b, the variation range in maximum daily GSR for both datasets for all four stations is 0.09–0.71 kWhm^−2^ day^−1^ with similar over-estimation situation for the Forest climatic zone. Meanwhile, the variation range in minimum daily GSR all showed under-estimation in the satellite datasets with the range of 0.57–0.9 kWhm^−2^ day^−1^. Under normal circumstances, satellite data rarely shows any exactness with ground measurements. However, considering that the reference GSR in this study was estimated and not a direct measurement, such slight variations are expected. For example, Sayago^18^ observed in comparing daily solar radiation retrieved from the NASA-POWER archives with ground measurements in Spain that, high coefficient of correlations was mainly associated with high solar radiation especially on clear days, and the opposite is true. Comparatively in this study, the best similarities are seen with the stations and seasons where high insolation occurs. Monteiro^20^ also reported good similarity of correlation coefficient and index of agreement to be 0.71 and 0.92 respectively when the NASA-POWER solar radiation datasets were compared with ground measurements over Brazil.

Meanwhile, in comparison with other studies which assessed a broader range of parameters including relative humidity, precipitation, and wind speeds, it is apparent that the NASA-POWER datasets show more consistency and accuracy with solar radiation related parameters^21^.

### Statistical analysis of the GSR_e_/GSR_n_ pairwise datasets

**Figure 4:**
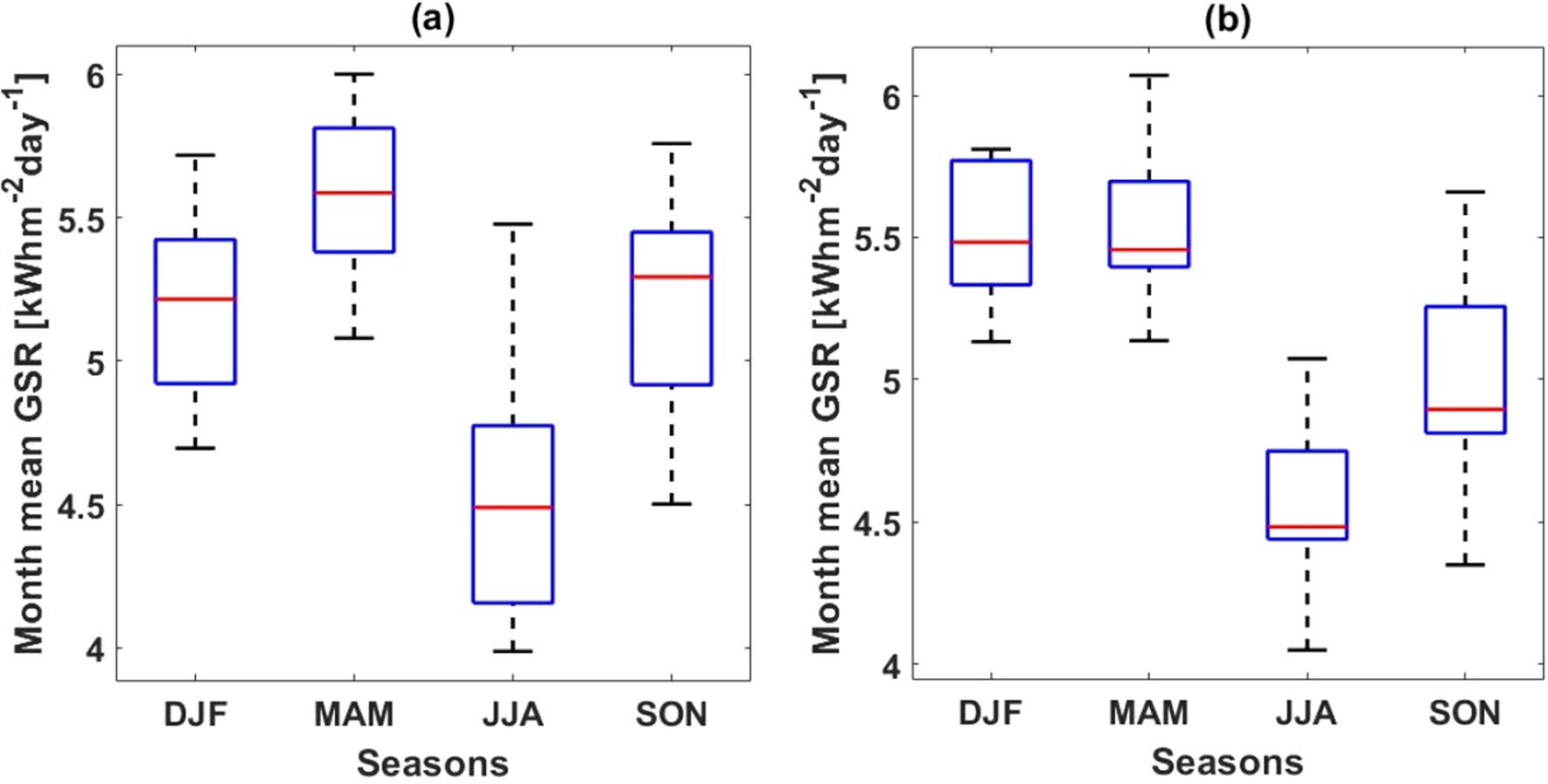
Boxplots showing seasonal variation in (**a**) estimated Global Solar Radiation (GSR_e_) and (**b**) NASA-POWER derived monthly mean GSR (GSR_n_). Seasons are December–January–February (DJF), March–April–May (MAM), June–July–August (JJA), and September–October–November (SON).

This section discussed various statistical approaches targeted at quantifying the strength of similarity between the GSR_e_/GSR_n_ pairwise datasets. Figure 4a and b show the boxplots for climatological monthly mean seasonal GSR_e_ and GSR_n_ datasets respectively. It is evident from the plots that, both GSR_e_ and GSR_n_ show semblance in inter-seasonal variation but some significant intra-seasonal variations. Inter-seasonally, both GSR_e_ (µ = 5.135 ± 0.08 kWhm^−2^ day^−1^) and GSR_n_ (µ = 5.137 ± 0.07 kWhm^−2^ day^−1^) show maximum insolation of 6.1 kWhm^−2^ day^−1^ and 6.2 kWhm^−2^ day^−1^ respectively during March–April–May (MAM) season hence only 0.78% deviation. For both datasets, peak GSR is in MAM. This inter-seasonal variation is obviously related to the annual migration of the ITCZ discussed in under the spatiotemporal comparison^23^. The seasonal variation in GSR distribution across the study area has been discussed by Asilevi^27^.

Intra-seasonally, the GSR_e_ and GSR_n_ datasets show remarkable differences in median and standard deviation. For example, each seasonal set in the GSR_n_ dataset show comparatively smaller interquartile ranges (IQR) of DJF = 0.44, MAM = 0.3, JJA = 0.31, and SON = 0.44 suggesting stronger relation within the satellite data than seasonal IQR in the GSR_e_ dataset of DJF = 0.5, MAM = 0.4, JJA = 0.62, and SON = 0.53. Again, the GSR_n_ dataset show relatively lower medians compared with the GSR_e_ datasets except of the DJF season, suggesting frequent under-estimations in the satellite data. These may be due to resolution and local climate specificity challenges with satellite observation data addressed by Dubovik^14^ and Kim^15^. Indeed similar variations were reported in the previous study by Asilevi et al.^27^ comparing estimated monthly mean GSR over Ghana with GSR data retrieved from the German Aerospace Centre (DLR) at a spatial resolution of 10 km by 10 km.

**Figure 5:**
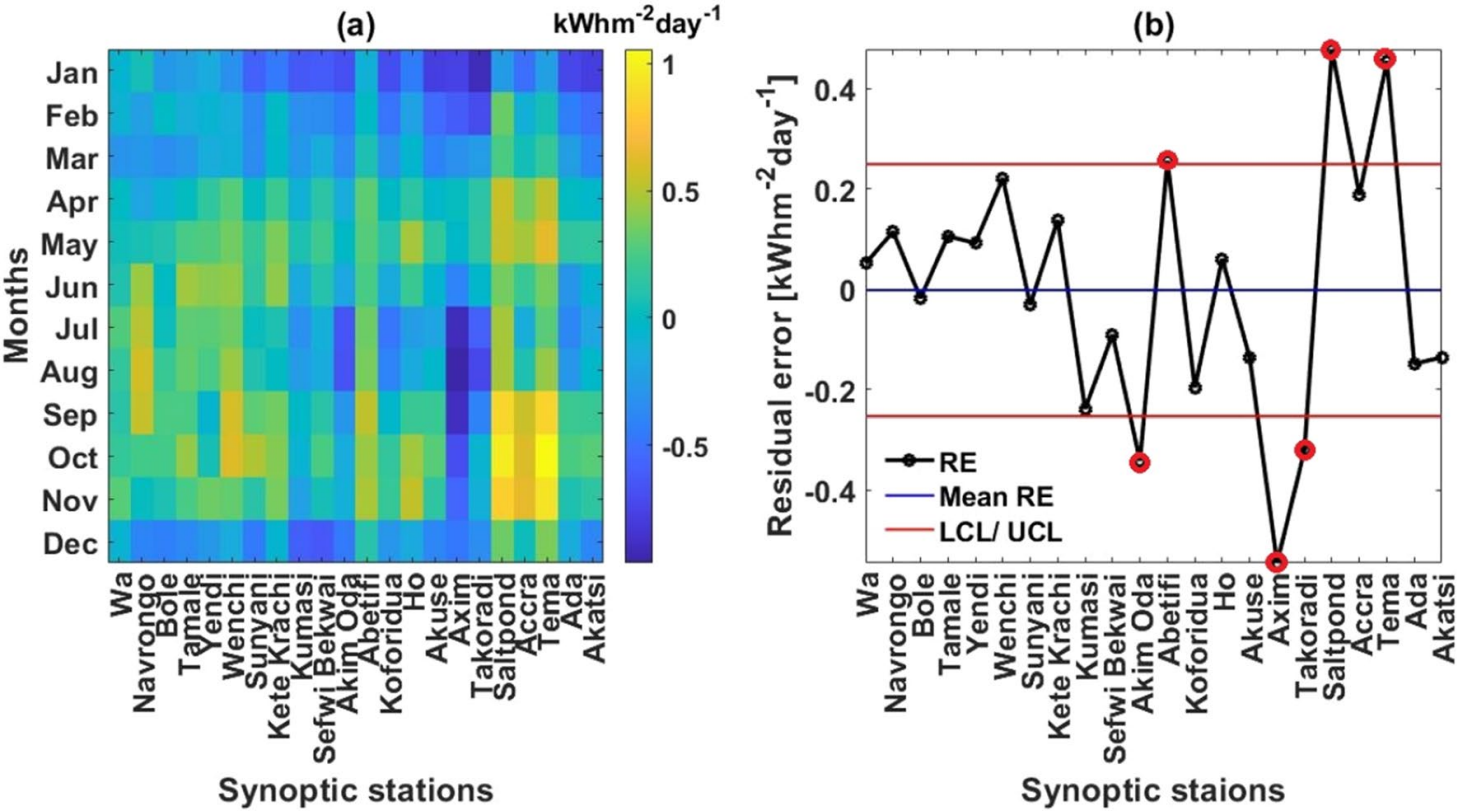
(**a**) Color plot depicting the climatological time and space variations of RE in the climatological GSR_e_/GSR_n_ pairwise datasets and (**b**) control chart depicting the station-by-station variation in mean climatological RE within the lower control limit (LCL) and upper control limit (UCL) of the standard deviation of RE.

To further analyze the pairwise datasets, the climatological residual errors (RE) between GSR_e_ and GSR_n_ datasets was calculated based on Eq. 4. The time and space variations of RE is shown in Fig. 5a. The absolute RE range in the pairwise datasets is 0.002–1.06 kWhm^−2^ day^−1^, with 51.1% positive REs and 48.9% negative REs indicating more higher GSR_e_ against GSR_n_ as depicted in Fig. 5a. The lowest absolute RE is in the northern half Savannah climatic zone during the MAM season whiles the highest is in the southern half Coastal climatic zone during the SON season, which can be attributed to the high and low atmospheric clarity during the MAM and SON seasons respectively, and the consequential high and low insolation in the respective seasons^27^. Additionally, the control chart in Fig. 5b shows that ~ 73% of station-by-station REs agree with the standard deviation of ± 0.25 around the mean RE, while only 27% is out of standard deviation range. This further demonstrates close semblance in the datasets.

**Figure 6:**
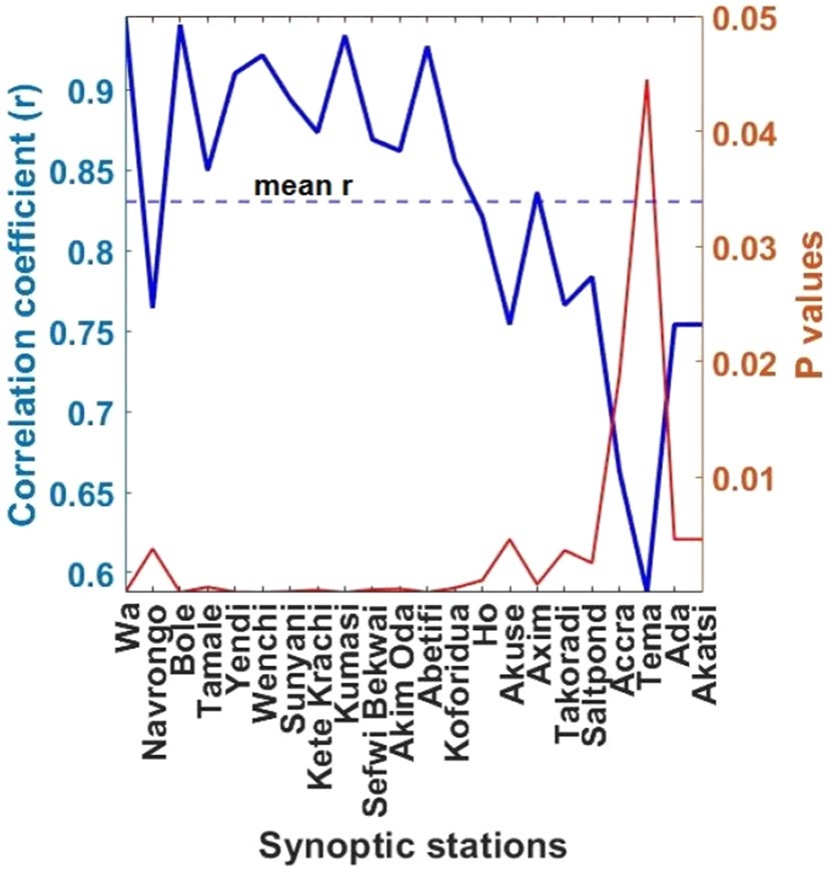
Station-by-station Pearson’s correlation coefficients (r) and their corresponding *p* values for the climatological GSR_e_/GSR_n_ pairwise datasets.

Figure 6 further shows an interesting trend in the Pearson’s correlation coefficients (r) comparing GSR_e_ and GSR_n_ with significant *p* values < 0.05. As seen, r decreases towards the coastal south of the study area where r values (0.59–0.75) are predominantly below the mean r = 0.83. Similar trend was reported in the previous comparison with the GSR data retrieved from the German Aerospace Centre (DLR) by Asilevi^27^. This has been attributed to the complex ocean–atmosphere–land interactions over the southern half, and the consequential frequent convective turbulences characterizing coastal tropics^37^. Undoubtedly, these conditions create significant satellite signal retrieval challenges. On the contrary, the northern half characterized by the highest atmospheric clarity indices of ~ 0.6 have a more stable atmosphere dynamics.

Table 3 summarizes all the statistical indices used in the comparisons. On the overall, the absolute ranges of RMSE = 0.13–0.46, MBE = 0.01–0.3, MPE = 1.11–6.34, r = 0.59–0.94, and d = 0.95–0.99 for all synoptic stations.

**Table 3:** Summary of statistical indices comparing estimated GSR and NASA POWER derived GSR for all stations in the four ecological zones. Root mean square error (RMSE), mean bias error (MBE), mean percentage error (MPE), Pearson’s correlation coefficient (r), and Willmott index of agreement (d).

Stations	RMSE	MBE	MPE	r	d
Wa	0.126	0.028	0.548	0.946	0.998
Navrongo	0.257	0.063	1.160	0.764	0.991
Bole	0.157	− 0.010	− 0.114	0.941	0.997
Tamale	0.219	0.058	1.088	0.850	0.993
Yendi	0.173	0.050	0.923	0.910	0.996
Wenchi	0.274	0.121	2.585	0.922	0.989
Sunyani	0.236	− 0.017	− 0.243	0.894	0.993
Kete Krachi	0.212	0.075	1.354	0.873	0.992
Kumasi	0.232	− 0.130	− 2.864	0.934	0.993
Sefwi Bekwai	0.224	− 0.049	− 1.144	0.869	0.993
Oda	0.303	− 0.186	− 4.259	0.862	0.982
Abetifi	0.234	0.140	2.708	0.927	0.986
Koforidua	0.248	− 0.107	− 2.407	0.856	0.987
Ho	0.229	0.033	0.488	0.821	0.989
Akuse	0.253	− 0.074	− 1.555	0.754	0.984
Axim	0.464	− 0.297	− 6.342	0.836	0.963
Takoradi	0.343	− 0.174	− 3.702	0.766	0.977
Salt Pond	0.423	0.261	4.967	0.784	0.968
Accra	0.284	0.102	1.830	0.663	0.977
Tema	0.426	0.248	4.476	0.588	0.953
Ada	0.238	− 0.081	− 1.655	0.754	0.989
Akatsi	0.253	− 0.074	− 1.555	0.754	0.984

Respectively in the order of Savannah, Transition, Forest, and Coastal zones, the mean absolute RMSE = 0.19, 0.24, 0.29, and 0.3, MBE = 0.04, 0.07, 0.15, and 0.13, MPE = 0.77, 1.39, 3.04, and 2.38, r = 0.88, 0.89, 0.84, and 0.69, and d = 0.99, 0.99, 0.98, and 0.97, depicting higher RMSE, MBE, and MPE over the southern half and lower over the northern half, while r and d are higher over the northern half and lower over the southern half.

## Conclusion

A statistical suitability assessment of the National Aeronautics and Space Administration—Prediction of Worldwide Energy Resources (NASA-POWER) satellite-derived solar radiation archives is presented. The NASA-POWER climatological datasets were compared with measured sunshine duration based estimated Global solar radiation (GSR) for 22 synoptic stations across four climatic zones in Ghana–West Africa. The results reveal that, the NASA-POWER monthly mean GSR (GSR_n_) ranging 3.69–6.15 ± 0.07 kWhm^−2^ day^−1^ showed good statistical semblance with sunshine duration based estimated GSR (GSR_e_) ranging 3.68–6.1 ± 0.08 kWhm^−2^ day^−1^ by correlation coefficient = 0.59–0.94, rmse = 0.13–0.46 and Willmott’s index of agreement = 0.95–0.99. Furthermore, both agree in zonal and seasonal GSR intensity distribution patterns with high and low zonal ranges in the Savanna (GSR_n_ = 4.46–6.11 ± 0.53 kWhm^−2^ day^−1^, GSR_e_ = 4.77–5.98 ± 0.4 kWhm^−2^ day^−1^) and Forest (GSR_n_ = 4.36–5.66 ± 0.48 kWhm^−2^ day^−1^, GSR_e_ = 4.11–5.57 ± 0.5 kWhm^−2^ day^−1^) respectively, and the peak and low seasonal ranges in March–May (GSR_n_ = 6. 1 kWhm^−2^ day^−1^, GSR_e_ = 6 kWhm^−2^ day^−1^) and June–August (GSR_n_ = 5.1 kWhm^−2^ day^−1^, GSR_e_ = 5.4 kWhm^−2^ day^−1^) respectively. It is expected that, as the results have shown good agreement between the estimated and satellite derived datasets, the NASA-POWER archives can be used extensively to develop a comprehensive solar energy resource assessment for the effective application of solar power systems and integration into the national grid. The results hereby provides empirical framework to develop a support system for the solar energy resource assessment task in the sub-region.

## Data Availability

The datasets used and/or analyzed during the current study are available from the corresponding author on reasonable request.
